# Formation of Wrinkled Nanostructures via Surface–Bulk Curing Disparity in Ethyl Cyanoacrylate: Toward Superhydrophobic Surface Applications

**DOI:** 10.3390/nano15010012

**Published:** 2024-12-25

**Authors:** Changwoo Lee, Heon-Ju Choi, Kyungeun Jeong, Kyungjun Lee, Handong Cho

**Affiliations:** 1 Pohang Institute of Metal Industry Advancement, Pohang-si 37666, Gyeongsangbuk-do, Republic of Korea; cw82.lee@pomia.or.kr; 2 School of Mechanical and Ocean Engineering, Mokpo National University, Muan-gun 58554, Jeollanam-do, Republic of Korea; gjswn2782@mokpo.ac.kr; 3 Department of Mechanical Engineering, Gachon University, Seongnam-si 13120, Gyeonggi-do, Republic of Korea; kecheong@gachon.ac.kr

**Keywords:** superhydrophobic surface, ethyl cyanoacrylate (ECA), wrinkled structure, abrasion resistance

## Abstract

Superhydrophobic surfaces, known for their exceptional water-repellent properties with contact angles exceeding 150°, are highly regarded for their effectiveness in applications including self-cleaning, antifouling, and ice prevention. However, the structural fragility and weak durability of conventional coating limit their long-term use. In this research, a new approach is proposed for the fabrication of long-lasting superhydrophobic surfaces using ethyl cyanoacrylate (ECA) and a primer. The application of the primer creates a curing rate disparity between the surface and bulk of the ECA layer, resulting in the formation of wrinkled microstructures essential for achieving superhydrophobicity. The fabricated surfaces were further functionalized through plasma treatment and hydrophobic silane (OTS) coating, enhancing their water-repellent properties. This straightforward and scalable method produced surfaces with excellent superhydrophobicity and robust adhesion to substrates. Durability tests, including roller abrasion and microscratch evaluations, indicated that the wrinkled structure and strong substrate adhesion contributed to sustained performance even under mechanical stress. Additionally, mechanical properties were assessed through nanoindentation, demonstrating enhanced resistance to physical damage compared to conventional superhydrophobic coatings. This study highlights the potential of ECA-based superhydrophobic surfaces for applications requiring durability and mechanical stability, such as architectural coatings, automotive exteriors, and medical devices. The approach offers a promising solution to the limitations of existing superhydrophobic technologies and opens new avenues for further research into wear-resistant and environmentally resilient coatings.

## 1. Introduction

Superhydrophobic surfaces, exhibiting a water contact angle of 150° or more, were developed by mimicking the Lotus effect, enabling the easy detachment and rolling of water droplets [1,2,3]. This distinctive characteristic results from the integration of hierarchical micro- and nanoscale surface structures with low-surface-energy materials and has been utilized across various industries for applications such as self-cleaning [4,5], fouling resistance [6,7,8], ice prevention [9,10], antifogging uses [11,12], corrosion resistance [13], and separation of oil and water [14,15]. However, superhydrophobic surfaces face several limitations in practical applications. Firstly, microstructures are susceptible to damage from external physical impacts or friction, which can degrade the surface’s hydrophobicity and cause it to lose functionality [16]. This vulnerability results in low durability, making long-term use challenging. Additionally, conventional superhydrophobic coatings are often applicable only to specific materials or are challenging to apply to substrates with complex shapes [17], thus limiting their range of applications.

To overcome these challenges, various studies have been conducted, including the development of coating matrices with strong bonding strength [18], enhancement of wear resistance through multilayer structures [19], incorporation of self-healing functionalities in coatings [20,21], and strengthening of interfacial adhesion using adhesives [22]. Among these, superhydrophobic surfaces utilizing ethyl cyanoacrylate (ECA), the main component of instant adhesives, have attracted significant attention. ECA can strongly adhere to various substrates and form adhesive layers with rapid and excellent bonding strength through polymerization in the presence of moisture [23]. However, previous studies faced limitations in fabricating superhydrophobic surfaces using ECA. Xin Du et al. proposed a simple process of applying 2-octyl cyanoacrylate monomer onto a substrate and immersing it in an aqueous ethanol solution to create a porous-structured superhydrophobic coating [24]. Despite its effectiveness, this method had constraints such as the high cost of 2-octyl cyanoacrylate, the requirement of a thin ECA coating thickness to form the porous structure, and the necessity of an inert atmosphere due to rapid reactions with moisture in the air. Moreover, common ECAs with higher surface energy than 2-octyl cyanoacrylate could not achieve contact angles exceeding 150°, limiting the complete realization of superhydrophobicity. In another study, Hui-Di Wang et al. presented a method to improve wear resistance of superhydrophobic coatings by using ECA as an adhesive to strengthen the adhesion between the substrate and the coating layer and forming a composite coating layer that includes PDMS and SiO_2_ nanoparticles [25]. However, challenges such as difficulty in uniformly dispersing nanoparticles, poor miscibility between ECA and PDMS, and the need for precise control of each component’s composition ratio could hinder the practical uses for superhydrophobic surfaces.

In this study, we address these issues by spraying a primer onto the surface of ECA to accelerate its curing speed, forming a highly rough wrinkled structure due to the difference in curing degrees between the surface and the interior of the ECA. Subsequently, we coated this wrinkled structure with a hydrophobic material to a superhydrophobic surface featuring a contact angle that exceeds 150°. This method allows for the creation of significant surface roughness without the use of nanoparticles and overcomes the limitations posed by the relatively high surface energy of ECA through hydrophobic coating. The fabricated superhydrophobic surface demonstrated excellent mechanical stability and durability, showing high adhesion and hardness in scratch and indentation tests. Furthermore, it maintained outstanding hydrophobic properties even after wear tests, proving its high potential for practical applications. This study presents a new approach to fabricating superhydrophobic surfaces using ECA, aiming to achieve technological advancements for practical implementation.

## 2. Materials and Methods

### 2.1. Materials

This study used the following reagents to fabricate ethyl cyanoacrylate (ECA)-based superhydrophobic surfaces. ECA was utilized in the form of an instant adhesive (Loctite 495, Henkel, Düsseldorf, Germany), and a primer (Loctite SF770, Henkel, Düsseldorf, Germany) was applied to accelerate the rapid curing of ECA. For the hydrophobic surface coating, n-octadecyltrichlorosilane (OTS, 95%, Thermo Fisher Scientific, Waltham, MA, USA) was used, and n-hexane (99.5%, Duksan Pure Chemical Co., Jincheon-gun, Republic of Korea) served as the solvent for diluting the OTS solution and for washing. All reagents were used as received from the manufacturers without any further purification.

### 2.2. Preparation of the Superhydrophobic Wrinkled ECA Surfaces

In this study, an instant adhesive (Loctite 495, Henkel, Düsseldorf, Germany) was thickly applied onto a glass slide to fabricate a superhydrophobic surface based on ECA. Immediately afterward, a primer (Loctite SF770, Henkel, Düsseldorf, Germany) was evenly sprayed over the ECA layer. The sample was then left in ambient conditions to allow the complete curing reaction to occur. The cured sample underwent plasma treatment for 3 min at 100 W using a low-frequency plasma generator (COVANCE-RF, Femto-Science, Hwaseong-si, Republic of Korea), introducing reactive functional groups onto the ECA surface. The plasma-treated sample was immersed for 10 min in an OTS solution diluted to a concentration of 0.1% *v*/*v* in n-hexane to perform the hydrophobic coating. After immersion, the sample was washed with clean n-hexane to remove any residual OTS and then dried in an oven at 65 °C for 12 h to stably form the hydrophobic layer.

### 2.3. Characterizations

The surface morphology and composition were analyzed using a field-emission scanning electron microscope (SEM, Regulus8230, Hitachi, Tokyo, Japan). Samples were coated with platinum to ensure conductivity and observed under an accelerating voltage of 5 kV. Additionally, the chemical changes occurring at each stage of the superhydrophobic surface fabrication process were examined using attenuated total reflectance Fourier transform infrared spectroscopy (ATR-FTIR, Frontier, Perkin Elmer, Waltham, MA, USA). The hydrophobic properties of the fabricated surfaces were evaluated by measuring the water contact angle. Contact angle measurements were performed using a droplet shape analyzer (SmartDrop, Femtobiomed, Seongnam, Republic of Korea). The contact angles were measured at ten different locations on each surface, and the mean values were calculated. The roller abrasion test was conducted using a custom-built apparatus. A load of 0.97 kg was applied, and the roller was reciprocated at a speed of 9.04 cm/s during the abrasion test. After every 50 abrasion cycles, the sliding angle and rebound coefficient were measured. Each sample was tested under the same conditions, with measurements repeated 10 times for each sample. The average values were used as the final results. A high-speed camera (Chronos 2.1-HD Kron Technologies, Burnaby, BC, Canada) was utilized to measure the rebound ratio of water droplets, and the recorded videos were analyzed using ImageJ software (version 1.54j). Before and after the abrasion tests, surface morphology changes were thoroughly examined using a confocal laser microscope (LEXT OLS5100, Olympus, Tokyo, Japan). The fabricated superhydrophobic coating’s mechanical properties and adhesion strength were assessed using a nanoindenter and microscratch testing apparatus (MCT3, Anton Paar, Graz, Austria). For mechanical property evaluation, the elastic modulus was measured using a Berkovich diamond indenter tip, and the values were calculated in accordance with the Oliver–Pharr method, with a minimum of five measurements taken for each sample to determine the average values. The interfacial adhesion strength between the superhydrophobic coating and the substrate was evaluated using a conical Rockwell diamond indenter with a tip radius of 10 µm, applying progressively increasing loads ranging from 30 to 500 mN. The scratch tests were performed at a constant speed with a final sliding length of 3 mm.

## 3. Results and Discussion

**Figure 1:**
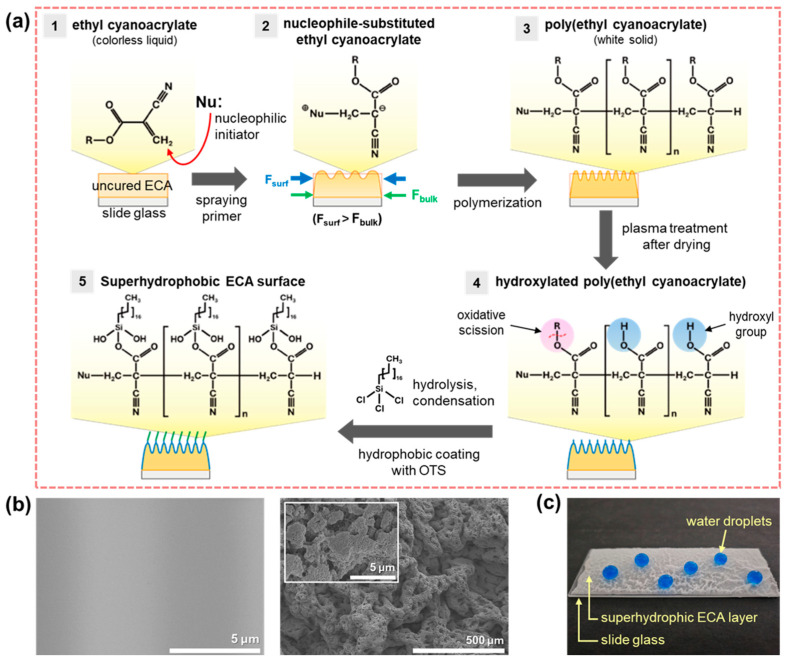
(**a**) Schematic illustration of wrinkle structure formation by curing ECA with primer application, (**b**) SEM images of the ECA surface cured without primer (**left**) and cured with primer application (**right**), and (**c**) photograph of the wrinkled ECA surface formed on a slide glass, exhibiting superhydrophobicity.

Superhydrophobic surfaces with wrinkled structures were fabricated by utilizing the disparity in bulk and surface polymerization rates of ethyl cyanoacrylate (ECA), as illustrated in Figure 1a. The primer for the instant adhesive accelerates the curing of ECA, introduces reactive functional groups to enhance adhesion strength, and expands the range of compatible substrates. This primer contains aliphatic amines [26], which act as nucleophilic initiators for ECA polymerization, triggering an exothermic reaction that rapidly produces high-molecular-weight polymers [27]. As shown in Figure 1a, primer is applied onto uncured ECA (Step 1). The aliphatic amines generate a large number of zwitterions on the ECA surface, which initiate polymerization. These zwitterions react with additional ECA monomers to propagate chain growth, transitioning the material from a transparent liquid to a solid white polymer (Step 2). Due to the high concentration of initiators on the surface, polymerization proceeds much faster at the surface than in the bulk. This results in significant contraction forces on the highly polymerized surface layer, while weaker contraction forces develop in the less-polymerized interior. The imbalance in these forces leads to the formation of large-scale wrinkled structures on the ECA surface (Figure 1b). Due to ECA’s excellent adhesion properties, the wrinkled structures consist of porous nanoscale features and strongly adhere to the substrate. ECA contains ethyl groups (-CH_2_CH_3_) that, when incorporated into the polymer chain, enhance hydrophobicity. However, the cyano groups (-C≡N) in ECA introduce limited hydrophilicity due to their polar nature [28], reducing the overall hydrophobicity. Thus, additional surface modifications are necessary to lower the surface energy. During polymerization, the double bonds (C=C) in ECA decrease, forming saturated repeating units and leaving fewer reactive sites for nucleophilic attack (Step 3). Furthermore, poly(ethyl cyanoacrylate) (PECA) exhibits high density and molecular weight, making it challenging for chemicals to penetrate the interior. To overcome this limitation, plasma treatment was employed to introduce reactive functional groups, such as hydroxyl (-OH) and carboxyl (-COOH), onto the PECA surface [29]. These groups enabled subsequent coating with hydrophobic materials to significantly enhance the surface’s hydrophobicity (Step 4). This study used n-octadecyltrichlorosilane (OTS), which features long alkyl chains, as the hydrophobic coating material [30]. During the coating process, the silane groups in OTS underwent hydrolysis reactions with trace amounts of water to form silanol (Si–OH) groups. These silanol groups reacted with the hydroxyl groups introduced during plasma treatment, forming stable siloxane (Si–O–Si) bonds that chemically anchored the OTS molecules to the surface. As the surface dried, the long alkyl chains self-assembled into a uniform hydrophobic layer, significantly reducing the surface energy and achieving superhydrophobicity (Step 5). As illustrated in Figure 1c, this method successfully fabricated a superhydrophobic surface on a glass slide. The polymerization of ECA resulted in a highly rough wrinkled structure, while subsequent chemical treatments enhanced the surface’s uniform superhydrophobicity. The proposed coating method effectively utilizes ECA’s excellent adhesion properties to anchor the wrinkled structures firmly to the substrate while minimizing the water contact area. Durable superhydrophobic surfaces were fabricated through additional chemical modifications, demonstrating potential for practical applications.

**Figure 2:**
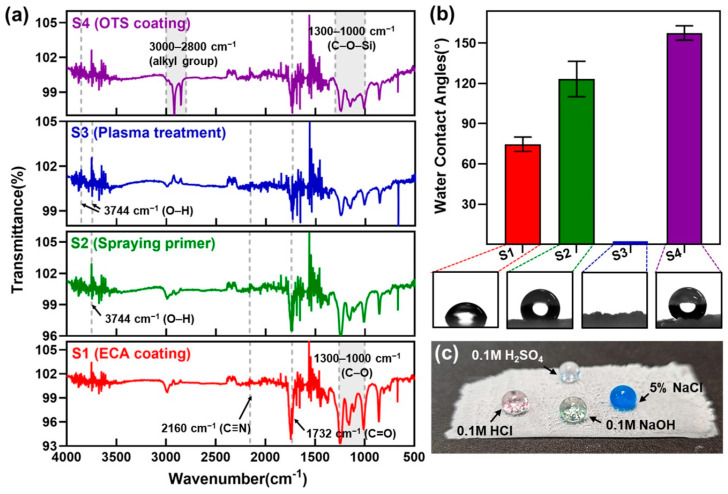
(**a**) ATR-FTIR spectra results related to the chemical changes during the fabrication of the superhydrophobic ECA surface, (**b**) static water contact angles and corresponding water droplet images for samples at different fabrication stages, and (**c**) photographs of droplets of 0.1 M HCl, 0.1 M NaOH, 0.1 M H_2_SO_4_, and 5% NaOH solutions resting on the superhydrophobic ECA surface.

ATR-FTIR analysis was performed on the sample surfaces at each coating stage to monitor the key functional groups’ presence and chemical changes during the fabrication of superhydrophobic surfaces. As shown in Figure 2a, after the application of ECA, distinct peaks at 1732 cm^−1^ and 2160 cm^−1^ were observed, corresponding to ester (-COOEt) and cyano (-C≡N) groups, respectively, indicating their introduction to the sample surface [31]. After applying the primer, a weak signal corresponding to hydroxyl (-OH) groups appeared at 3744 cm^−1^, but minimal changes were observed at 1732 cm^−1^ (C=O) and 2160 cm^−1^ (C≡N). This suggests that the aliphatic amine in the primer reacted with the cyano or ester groups on the ECA surface to generate a small amount of hydroxyl groups [27]. Following plasma treatment, distinct peaks were observed at 3744 cm^−1^ and 3855 cm^−1^, corresponding to hydroxyl groups, along with intensity changes in the peaks associated with carbonyl and cyano groups. These changes suggest that oxidation occurred during the plasma treatment process [32]. Although a significant increase in hydroxyl groups was expected, the ATR-FTIR results showed otherwise. This is likely due to the redistribution of hydroxyl groups from the surface to the polymer’s interior over time, as the ATR-FTIR measurements were not conducted immediately after plasma treatment. Finally, after OTS coating, trichlorosilane groups reacted with the hydroxyl groups on the surface to generate stable Si–O–Si siloxane bonds. This reaction was confirmed by an increased signal in the 1000–1300 cm^−1^ range corresponding to C–O–Si bonds. Additionally, the introduction of the octadecyl (C_18_) alkyl chains from OTS enhanced the signal in the 2800–3000 cm^−1^ range, representing alkyl groups [33]. The formation of siloxane bonds and the incorporation of long alkyl chains enabled the final superhydrophobicity on the ECA surface. Figure 2b shows the contact angle measurements at different fabrication stages. After ECA application and curing under ambient conditions, the contact angle was measured at 74.7°, indicating hydrophilic behavior. This suggests that the ethyl groups in ECA alone are insufficient to impart significant hydrophobicity. After primer application and complete curing, the contact angle increased to 122.6°. As evidenced by the ATR-FTIR results in Figure 2a, the slight increase in hydroxyl groups and the rough surface formed during curing likely contributed to enhanced hydrophobicity. When the contact angle was measured immediately after plasma treatment, the water droplet spread rapidly, indicating superhydrophilicity. This observation, combined with the ATR-FTIR data, suggests that plasma treatment introduced a significant number of hydroxyl groups to the ECA surface. However, over time, these hydroxyl groups likely migrated from the surface, emphasizing the importance of promptly applying the OTS coating after plasma treatment to ensure stable chemical bonding and the formation of a robust hydrophobic layer. After OTS coating, the contact angle dramatically increased to 157.6°, confirming superhydrophobicity. This enhancement can be attributed to the introduction of OTS’s long alkyl chains, which significantly reduced surface energy, and the synergistic effect of the surface’s wrinkled structure. The combination of these factors enabled the effective realization of superhydrophobic properties. To further explore environmental resistance, the chemical resistance of the superhydrophobic ECA surface was evaluated by placing droplets of 0.1 M HCl, 0.1 M NaOH, 0.1 M H_2_SO_4_, and 5% NaOH solutions on the surface. As shown in Figure 2c, the droplets retained their spherical shapes with no signs of wetting or spreading, demonstrating the surface’s robust hydrophobicity under both acidic and basic conditions. These results highlight the chemical stability of the ECA-based surface and its potential for applications in environments with exposure to extreme pH conditions.

**Figure 3:**
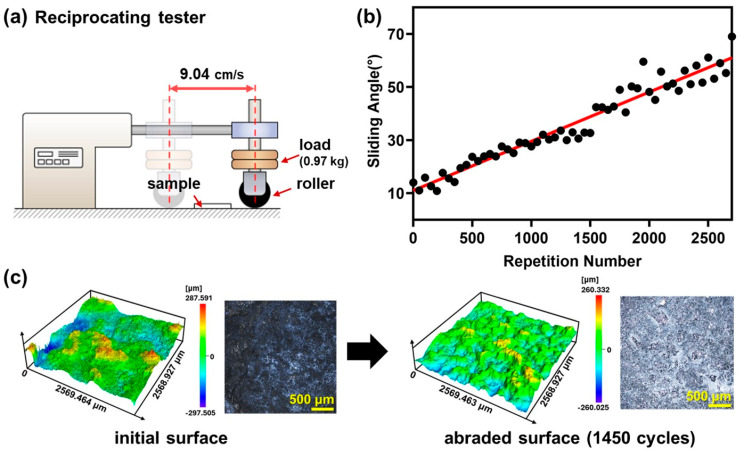
(**a**) Schematic illustration of the custom-built apparatus for the abrasion test, (**b**) sliding angles of water droplets after abrasion cycles (average of 10 measurements per sample), and (**c**) confocal microscopy images of the ECA surface before (**left**) and after (**right**) the abrasion test.

A roller abrasion test was conducted to assess the durability of superhydrophobic ethyl cyanoacrylate (ECA) surfaces with wrinkled structures, as illustrated in Figure 3. In this experiment, a roller was reciprocated over the sample surface under a constant load to induce abrasion, and the sliding angle of the abraded samples was measured to observe changes in surface wettability. The initial sliding angle was approximately 10°, indicating excellent superhydrophobic properties. However, after 2500 reciprocating cycles, the sliding angle gradually increased to about 60°. Despite the increase in sliding angle, the surface maintained a high static contact angle and exhibited a significant degree of hydrophobicity. Confocal microscopy was utilized to examine morphological changes in the abraded surface. The analysis revealed that the wrinkled structures on the surface were substantially preserved even after the abrasion test, maintaining high surface roughness. The preservation of the wrinkled structures was critical in minimizing the surface area in contact with the water droplet, thereby maintaining a high static contact angle even after abrasion. This durability is attributed to the unique mechanical properties of the highly cross-linked polymer network formed during the curing process of ECA. The cured ECA exhibits high hardness and density, which significantly improve its resistance to physical damage during abrasion testing. In contrast, the OTS coating layer exhibits significantly lower mechanical strength compared to ECA and is susceptible to removal during abrasion. The removal of the OTS coating exposes the underlying ECA, resulting in localized adhesion of water droplets to the surface and an increased sliding angle.

**Figure 4:**
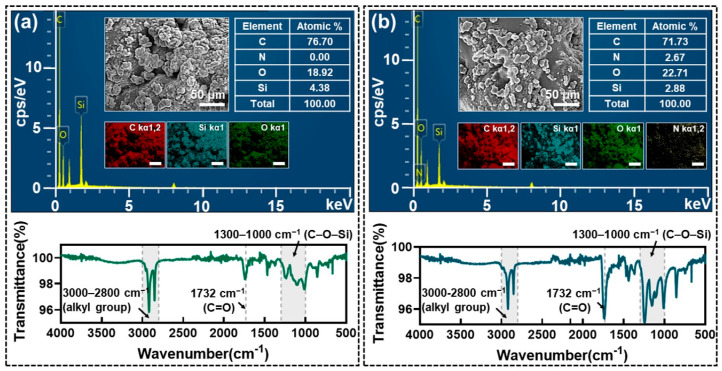
Characterization of the superhydrophobic ECA surface: (**a**) SEM, EDS, and FT-IR results before sandpaper abrasion and (**b**) corresponding results after sandpaper abrasion.

To explore the effects of more aggressive abrasion compared to the roller test, the superhydrophobic ECA surface was examined by subjecting the surface to 30 cycles of rubbing with 240 g sandpaper under a controlled load. The surface morphology, elemental composition, and chemical changes were analyzed using SEM, EDS, and FT-IR, respectively, with results displayed in Figure 4. Figure 4a presents the results of the ECA surface before abrasion. SEM images reveal the characteristic wrinkled structure of the surface, which contributes significantly to its superhydrophobic properties. EDS analysis shows that nitrogen content was undetectable, which is consistent with the composition of the OTS coating layer, primarily containing elements like silicon and carbon. FT-IR spectra exhibit strong peaks corresponding to alkyl groups (3000–2800 cm^−1^) and silane groups (1300–1000 cm^−1^), confirming the presence of the hydrophobic OTS coating. Figure 4b illustrates the corresponding results after the abrasion test. SEM images indicate that the wrinkled structures were significantly smoothed, leading to a loss of surface roughness. EDS analysis shows an increase in nitrogen content, attributed to the removal of the OTS coating and the subsequent exposure of the underlying ECA, which contains nitrogen-rich cyano groups. FTIR spectra demonstrate a decrease in the peaks corresponding to alkyl groups (3000–2800 cm^−1^) and silane groups (1300–1000 cm^−1^), indicating the partial removal of the hydrophobic OTS coating. Despite these changes, FT-IR spectra still detect residual alkyl groups, suggesting that portions of the hydrophobic layer remained in the valleys of the wrinkled structures, which were less affected by abrasion. EDS analysis further shows that carbon remains the dominant surface element post-abrasion, supporting the retention of hydrophobicity. This indicates that even after the removal of the OTS layer, the exposed ECA contributes to the surface’s hydrophobicity due to its carbon-rich composition. The combined SEM, EDS, and FTIR analyses suggest that the wrinkled ECA surface, even after the removal of the OTS coating layer from exposed regions, maintains considerable hydrophobicity due to the synergistic effect of the chemical properties and the inherent mechanical strength of the ECA material.

**Figure 5:**
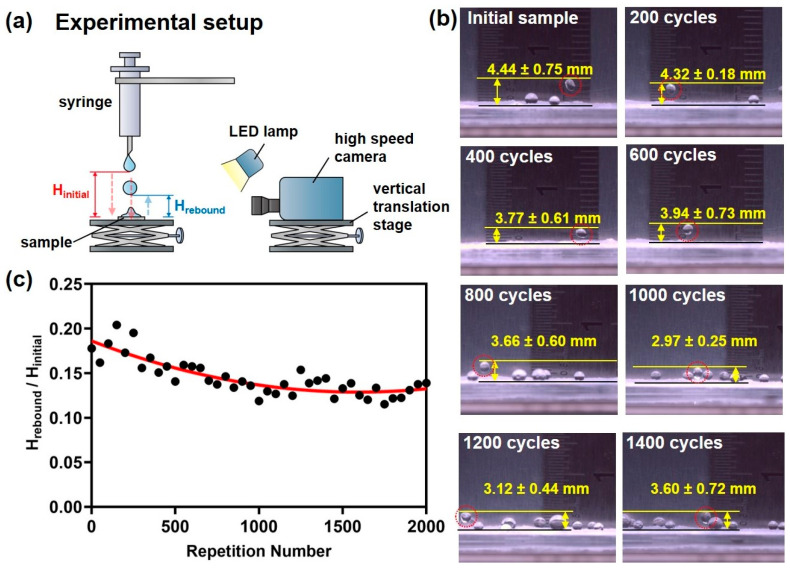
(**a**) Experimental setup for measuring the water droplet rebound ratio, (**b**) high-speed camera images capturing water droplet rebound on abraded surfaces, and (**c**) rebound ratio results for abraded surfaces (average of 10 measurements per sample).

Furthermore, as illustrated in Figure 5a, water droplet rebound tests were conducted on the abraded surface. In the water droplet rebound test, a droplet was released from a fixed height onto the sample surface, and the rebound height was measured using a high-speed camera. By calculating the ratio of the rebound height to the initial drop height (rebound ratio), we evaluated the initial hydrophobic properties and the ability to maintain hydrophobicity after abrasion. The rebound ratio of water droplets on the ECA-based superhydrophobic surface before abrasion was approximately 0.2. Changes in the rebound ratio were measured as the surface underwent up to 2000 abrasion cycles. As the number of abrasion cycles increased, the rebound ratio gradually decreased, reaching about 0.13 after 1000 cycles and remaining stable up to 2000 cycles. Even after extensive abrasion, the water droplets did not penetrate the surface but instead bounced off, demonstrating the sustained hydrophobicity. These results indicate that the wrinkled structures formed by ECA contribute to maintaining an air layer even after repeated abrasion tests, effectively reducing the contact area between water droplets and the surface, thereby allowing hydrophobicity to persist. Therefore, ECA-based superhydrophobic surfaces possess not only excellent initial hydrophobic properties but also outstanding durability under repetitive mechanical abrasion conditions. This enhances their potential for applications in fields requiring high durability, such as architectural exterior materials, antifouling coatings, and self-cleaning surfaces.

**Figure 6:**
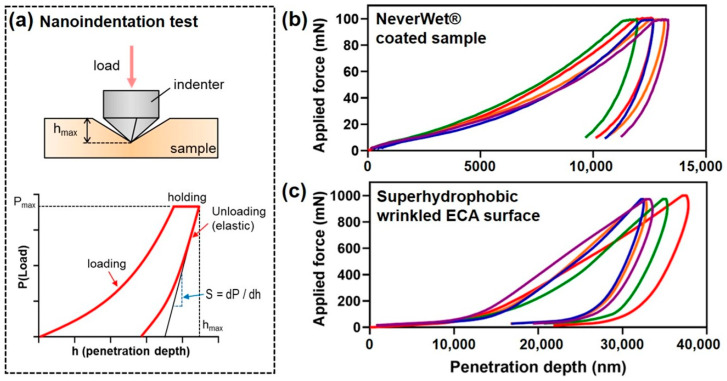
(**a**) Illustration of nanoindentation for measuring the elastic modulus through nanoscale load and displacement, (**b**) load–displacement curve of the surface coated with a commercial superhydrophobic coating (NeverWet^®^), and (**c**) load–displacement curve of the wrinkled superhydrophobic ECA surface.

In this study, nanoindentation experiments were conducted to quantitatively evaluate the mechanical properties of superhydrophobic surfaces fabricated with ethyl cyanoacrylate (ECA) (Figure 6). The durability and mechanical strength of ECA-based superhydrophobic surfaces were evaluated and compared to superhydrophobic surfaces fabricated using the commercially available coating NeverWet^®^ (RustOleum Corp., Vernon Hills, IL, USA) to assess their practical applicability. Superhydrophobic surfaces are typically characterized by their water contact angles, but their practical utility in real-world applications is closely related to their resistance to external loads, friction, and impacts. Therefore, elastic modulus is a critical mechanical property that determines the durability of superhydrophobic surfaces [9]. Nanoindentation experiments were performed to evaluate these properties quantitatively. Nanoindentation is a precise technique for determining the elastic modulus and hardness of materials by measuring the load and displacement at the nanoscale, as illustrated in Figure 5a. The Oliver–Pharr method was applied to calculate the elastic modulus, which involves deriving the reduced modulus (*E_r_*) from the slope (*S*) of the unloading curve in the load-displacement data and the contact area (*A*). The formula for the Oliver–Pharr method is as follows [34,35]:

(1) $$E_{r}=\frac{1}{2}\cdot \frac{\sqrt{\pi }}{\beta }\cdot \frac{S}{\sqrt{A}}$$

where *β* is the shape factor of the indenter. The reduced modulus *E_r_* integrates the elastic moduli of both the sample and the indenter and is expressed as

(2) $$\frac{1}{E_{r}}=\frac{1-\nu^{2}}{E}+\frac{1-\nu_{i}^{2}}{E_{i}}$$

Here, *E* and *E_i_* are the elastic moduli of the sample and the indenter, respectively, while *ν* and *ν_i_* are their Poisson’s ratios. In this study, the indenter was made of diamond, a highly rigid material, allowing the deformation of the indenter to be neglected. Consequently, the reduced modulus (*E_r_*) was approximated as the elastic modulus (*E*) of the sample. NeverWet^®^ is a commercially available superhydrophobic coating consisting of a two-part system: an adhesive base coat and a superhydrophobic top coat. The base coat ensures strong adhesion to the substrate, while the top coat, made of silica nanoparticles and polymer binders, reduces surface energy to achieve superhydrophobicity. This dual-layer structure facilitates high water contact angles, providing effective water repellency [33,36]. Although NeverWet^®^ achieves superhydrophobicity through high contact angles, its low mechanical strength makes it susceptible to damage from external forces or friction. The experimental results showed that the average reduced modulus of the NeverWet^®^ coating was 1.307 GPa (Figure 6b), indicating weak molecular interactions and low resistance to external loads. This low mechanical strength limits the coating’s durability, affecting its longevity in practical applications. In contrast, the ECA-based superhydrophobic surface exhibited an average reduced modulus of 4.595 GPa (Figure 6c), approximately 3.5 times higher than that of the NeverWet^®^ coating. During the curing process, ECA forms a highly cross-linked three-dimensional network structure with strong polymer chain bonding, resulting in high density and mechanical strength. The differences in elastic modulus between the ECA and NeverWet^®^ coatings stem from their material composition and structural properties. While NeverWet^®^ offers the advantage of easily achieving superhydrophobicity, its low mechanical strength limits its durability in demanding environments. In contrast, the ECA-based superhydrophobic surface exhibits a high elastic modulus and excellent durability, providing exceptional resistance to physical damage. The nanoindentation results confirm that ECA-based superhydrophobic surfaces possess significantly superior mechanical properties compared to the commercial NeverWet^®^ coating.

**Figure 7:**
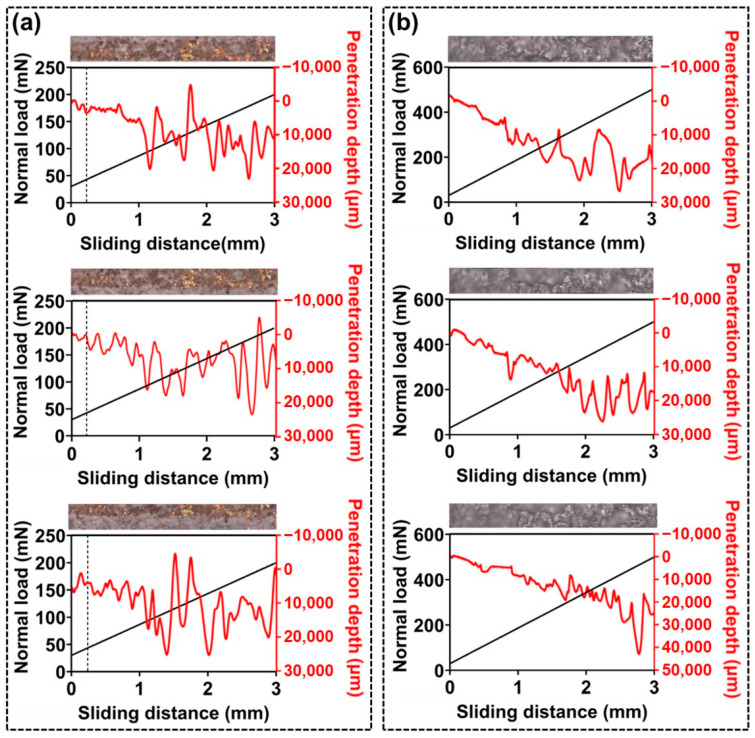
(**a**) Optical micrographs showing initial surface failure during scratch tests for NeverWet^®^-coated surfaces and (**b**) corresponding results for superhydrophobic wrinkled ECA surfaces.

As shown in Figure 7, in addition to nanoindentation experiments, microscratch tests were conducted to evaluate the durability of ECA-based superhydrophobic coating layers. The microscratch test is used to assess the adhesion strength between the coating layer and the substrate by measuring the load at which delamination occurs while sliding a diamond indenter over a fixed distance under progressively increasing loads [37]. This test provides crucial information on how well the coating layer can withstand external friction or impact. In this study, the durability of the ECA coating layer was quantitatively analyzed in comparison with that of the NeverWet^®^ coating. As observed in Figure 7a, delamination of the NeverWet^®^ coating layer began at a load of approximately 42 mN at the interface with the substrate. This indicates that the NeverWet^®^ coating layer has not only low mechanical strength but also weak adhesion to the substrate. The weak bonding between the inner adhesive layer and the outer superhydrophobic layer in the dual-layer structure of NeverWet^®^ may contribute to this delamination phenomenon. In contrast, the superhydrophobic coating layer fabricated with ECA showed no delamination even when the load was increased up to 500 mN during the microscratch test. This clearly demonstrates that the ECA coating layer is very strongly bonded to the substrate. This result is attributed to the unique chemical properties and curing mechanism of ECA. During the curing process, ECA forms strong chemical bonds with activated functional groups on the substrate surface and reinforces adhesion through a rigid three-dimensional network structure. Therefore, the ECA-based coating layer possesses much more robust characteristics against external loads or friction, and its durability in practical environments is expected to be excellent. These findings suggest that the structural characteristics of ECA-based coatings enhance durability and resistance to external loads, making them well suited for durability-critical applications across diverse industrial sectors.

## 4. Conclusions

In this study, we successfully fabricated superhydrophobic surfaces with wrinkled structures using ethyl cyanoacrylate (ECA) and a primer. The application of the primer induced a difference in curing rates between the ECA surface and the bulk, leading to the formation of highly rough wrinkled structures that played a key role in achieving superhydrophobicity. To enhance the inherently low hydrophobicity of ECA, hydroxyl groups were introduced onto the surface via plasma treatment, followed by the application of a hydrophobic silane (OTS) coating containing long alkyl chains, resulting in excellent superhydrophobic properties.

Durability assessments using roller abrasion tests confirmed that the ECA surface maintained its water-repellent properties even after significant abrasion, effectively preventing water penetration due to the persistent wrinkled structure and air layer. However, abrasion led to the removal of the OTS coating layer, exposing the underlying ECA, which increased the sliding angle and caused localized water adhesion. Additionally, mechanical testing through nanoindentation and microscratch tests demonstrated that the ECA surface exhibited a significantly higher elastic modulus and strong adhesion to the substrate compared to commercial coatings, highlighting its robustness and suitability for demanding environments.

Compared to traditional superhydrophobic surface fabrication methods, such as chemical etching or nanoparticle deposition, the proposed technique offers a simplified, cost-effective, and scalable alternative. These conventional methods often involve complex processes, high costs, limited substrate compatibility, and poor adhesion of nanoparticles, which restrict their practical applicability. In contrast, the ECA-based approach not only addresses these challenges but also provides superior mechanical durability, making it a strong candidate for applications in industries requiring high performance, such as wind turbine blades, automotive coatings, medical devices, and antifouling surfaces.

Future research will focus on enhancing the wear resistance of the hydrophobic silane layer and developing composite coating technologies capable of maintaining hydrophobicity under extensive mechanical and environmental stress. Furthermore, systematic evaluations under extreme conditions, including high temperatures and chemical corrosion, are necessary to fully understand and extend the practical applications of ECA-based superhydrophobic surfaces.

This study presents a novel and versatile superhydrophobic surface fabrication technique that leverages curing rate differences and wrinkled structure formation to overcome the limitations of existing coating technologies. By achieving enhanced durability, simplicity, and scalability, this technology demonstrates significant potential for adoption across various industrial sectors.

## Data Availability

The research data of this work are available upon request to authors.
